# Cytotoxicity and immunological impact of *Trypanosoma* sp. infection on blood parameters of wild African catfish, *Clarias gariepinus*

**DOI:** 10.1007/s00436-023-08026-8

**Published:** 2023-12-07

**Authors:** Alamira Marzouk Fouad, Rasha S. A. Abd El-Lateif, Haitham G. Abo-Al-Ela, Sara Salah Abdel-Hakeem

**Affiliations:** 1 Department of Aquatic Animal Medicine and Management, Faculty of Veterinary Medicine, Assiut University, Assiut, 71526 Egypt; 2 Unit of Fish Diseases and Management, Animal Health Research Institute (AHRI) Agriculture Research Center (ARC), Assiut Lab, Assiut, Egypt; 3 Genetics and Biotechnology, Department of Aquaculture, Faculty of Fish Resources, Suez University, Suez, 43221 Egypt; 4 Parasitology Lab., Zoology and Entomology Department, Faculty of Science, Assiut University, Assiut, 71526 Egypt

**Keywords:** Cytotoxicity, Erythrocytes, Fish health, Immune response, Parasitism, Trypanosome

## Abstract

Fish trypanosomiasis is a common blood parasitic disease transmitted by aquatic invertebrates, such as leeches. This study aims to shed light on the cytotoxicity of *Trypanosoma* sp. on erythrocytes and its impacts on the innate immune response (serum lysozyme activity, nitric oxide production, phagocytic activity, serum total protein, and globulin) in wild African catfish, *Clarias gariepinus*. One hundred catfish were examined using blood smears stained with Giemsa and confirmed with PCR. The prevalence of infection was found to be 10% by microscope detection and 15% by PCR. The morphological identification of *Trypanosoma* as *Trypanosoma mukasai* was determined. Additionally, this study included previously undescribed features of *Trypanosoma*, such as the width of the anterior and posterior body, the length of the posterior pale region, and the number of folds. Various alterations in erythrocytes were observed, totaling 54.57%. Nuclear abnormalities, including fragmented nuclei, eccentric nuclei, and micronuclei, were also reported. Infected fish showed a reduction in serum total protein and globulin levels, while nitric oxide production, lysozyme activity, and phagocytic activity exhibited a significant increase compared to non-infected fish. We believe that our findings will contribute valuable data to the morphological and molecular identification of *Trypanosoma* sp. in African catfish, as well as their cytotoxic impact.

## Introduction

Parasitic infections remain a significant public health concern, posing various risks to the biological aspects of fish populations and causing pathological damage to both wild and farmed fish (Abdel-Hakeem et al. 2019; Mahmoud et al. 2020). However, there is limited information available regarding diseases caused by hemoparasites and their impact on fish health, particularly concerning parasites belonging to the *Trypanosoma* genus (Lourenço et al. 2014). Trypanosomes are extracellular blood-borne parasites found in different species of marine and freshwater fish (Hayes et al. 2014). Based on comparative analyses, it has been suggested that these parasites undergo development from free-living aquatic forms to aquatic invertebrates such as annelids and leeches, and are subsequently transmitted to aquatic vertebrates, including fish and amphibians (O'Donoghue 2017).

Approximately 200 species of *Trypanosoma* have been identified in fish based on phylogenetic, morphological, host-specific, and geographical characteristics (Ferreira and Avenant-Oldewage 2013). These *Trypanosoma* are distinguished by the presence of a flagella that grows from a basal body called kinetoplast, and they reproduce asexually through longitudinal binary fission (Hoffman 1999). In infected fish blood, trypanosomatids undergo various morphological transformations, including amastigote, promastigote, epimastigote, and trypomastigote forms. Fish blood trypomastigotes are transformed into slender epimastigotes inside the leeches' gut. Once they have become metacyclic trypomastigotes, the parasites migrate to the mouthparts where they can be injected into the area around the proboscis of the next host during the subsequent blood meal (Eiras et al. 2008; Molina et al. 2016).

However, descriptions of fish *Trypanosoma* spp. have solely relied on morphology and host taxonomy criteria (Eiras et al. 2012). These criteria are insufficient in cases of mixed trypanosome infections, which have been detected through molecular studies (Grybchuk-Ieremenko et al. 2014). As a result, the diagnosis of trypanosome infections may rely on clinical signs, parasitological examination, serological techniques, and molecular methods such as the PCR technique, which is theoretically highly sensitive and specific (Eisler et al. 2004).

Blood cells have been utilized as markers of toxicity and effective tools for examining the effects of fish exposure (Esmaeili 2021; Mekkawy et al. 2011; Sayed 2018). Furthermore, trypanosome infections, similar to other hemoflagellates, can alter the physicochemical characteristics of their hosts (Jones 2001). Therefore, blood parameters are crucial for assessing the impact of trypanosome infections on fish homeostasis (Shah and Altindag 2004). One important parameter for assessment is the detection of phagocytic activity. Phagocytosis in the blood plays a vital role in preventing infectious diseases, involving the internalization and elimination of harmful organisms (Panigrahi et al. 2005). Phagocytes can be activated by opsonin-triggered serum lysozymes (Magnadóttir 2006), which are commonly used to evaluate innate immunity in fish (Tort et al. 2003). Additionally, nitric oxide (NO) is a crucial effector of the immune system in reducing and eliminating various microorganisms (Gobert et al. 2000). Moreover, the estimation of serum total proteins and globulins can provide indications of liver function in the host. In the case of trypanosome infection, the liver may lose its functionality, leading to decreased levels of serum total proteins and globulins (Aly et al. 2005).

*Clarias gariepinus*, a member of the *Clariidae* family, was introduced worldwide in the early 1980s for aquaculture purposes. It is one of the most commercially important freshwater fish in many developing countries in Africa (Ferreira and Avenant-Oldewage 2013). This fish species has experienced significant geographical spread, possesses a high growth rate, can tolerate high stocking densities, is well-accepted by consumers, and exhibits high resistance to poor water quality and oxygen depletion (Karami et al. 2010). Furthermore, due to its well-documented biology, it is frequently employed in fundamental research and is regarded as an excellent model for toxicological studies (Sayed et al. 2013).

The pathogenesis and cytotoxicity of fish trypanosomiasis on erythrocytes are not well understood. However, studies have reported changes in hematological parameters (Eiras et al. 2008; Ferreira and Avenant-Oldewage 2013; Fujimoto et al. 2013). There is a lack of literature on *Trypanosoma* spp. in *C. gariepinus* in Egypt, as well as the associated immune responses following infection. Therefore, the current study was to investigate cytotoxic effects of the parasite on erythrocytes and its impact on the immunomodulation of non-specific innate immunity. To the best of our knowledge, this is the first study to explore the cytotoxic effects of *Trypanosoma* sp. and the immune responses of *C. gariepinus*.

## Materials and methods

### Animal sampling

One hundred specimens of *C. gariepinus*, weighing between 300–450 g and measuring 35–42 cm in total length, were collected from the Nile River in Assiut Governorate, Egypt. The collection site was located at Latitude 12 4.229' N and Longitude 10′48.639' E. Sampling took place from February to May 2021, twice a week. The specimens were immediately transported alive to the Parasitological Laboratory in the Department of Zoology and Entomology, Faculty of Science, Assiut University, Egypt, using a plastic aquarium for further analysis.

The specimens were examined based on the morphological features described by Willoughby (1974). Measurements including total length, standard length, and body weight were assessed for each specimen.

### Clinical examination

All fish were initially examined for any visible external abnormalities. Following that, they were incised to observe and document any internal postmortem signs that were detected.

### Parasitological examination

Blood samples were collected from the caudal vein of live specimens. A portion of the collected blood samples was utilized to create thin blood smears, with ten slides prepared per sample. The blood films were then air-dried at room temperature and fixed in absolute methanol. Subsequently, staining was performed using Giemsa stains, hematoxylin, and eosin (Hayes et al. 2006). The stained slides were examined under a light microscope with objective lenses of 40 × and 100 × magnification (Optika B-500Ti, Italy) to identify any present parasites.

Another portion of the blood samples was allocated for DNA extraction. The remaining blood samples were subjected to centrifugation at 10,000 xg for 5 min to separate the blood cells from the serum. The serum was then utilized for immunological investigations. Microscopic identification of the parasites was performed following the freshwater parasite pictorial guide keys provided by Okoye Uzodinma et al. (2016). Photographs of the parasites were captured using a digital camera. Morphometric analysis was conducted using Motic Image Advanced 3.0 software, with all measurements provided in micrometers unless stated otherwise in the results section.

### Confirmation of *Trypanosoma* sp. using PCR

Total genomic DNA was extracted from concentrated fresh blood samples (Takeet et al. 2013) of all examined fish using the DNeasy Blood and Tissue Kit (Qiagen, Germany), following the manufacturer’s protocol. The purity and concentration of the DNA were measured using a nanophotometer (Implen GmbH, Germany) and stored at –20 °C until further use.

Next, PCR was conducted, following the method described by Maslov et al. (1996) with some modifications. Briefly, a COSMO PCR RED Master Mix kit (Willofort, UK) was used in a 50 µl reaction volume. 200 ng of DNA was mixed with 2X COSMO PCR RED Master Mix, and 20 pmol of each forward primer D (5'-ACCGTTTCGGCTTTTGTTGG-3') and reverse primer H (5'-CGTCAATTTCTTTAAGTTTC-3') to amplify the *Trypanosoma*-specific SSU rRNA gene. The amplification process involved the following conditions: an initial denaturation step at 95 °C for 5 min, followed by five cycles at 95 °C for 1 min, 45 °C for 30 s, and 65 °C for 1 min. Subsequently, 35 cycles were performed at 95 °C for 1 min, 60 °C for 30 s, and 72 °C for 1 min. The final extension was carried out at 65 °C for 10 min.

The PCR products were analyzed using 1.5% agarose gel in Tris–acetate-EDTA (TAE) buffer, stained with ethidium bromide (50 μl/L), and visualized under a UV transilluminator. The size of the PCR products was determined using the GeneRuler 100 bp DNA ladder (Thermo-Scientific, Germany).

### Cytotoxicity of erythrocytes

The cytotoxicity of *Trypanosoma* sp. was demonstrated by assessing the malformations of erythrocytes and nuclear abnormalities. A total of 10,000 cells (1,000 per slide) were examined in both infected and non-infected fish, using a 100X objective (Al-Sabti and Metcalfe 1995). The average number of malformed erythrocytes was determined in relation to the ratio of normal RBCs. To evaluate micronucleus occurrence, 3,000 erythrocytes per fish were observed at a magnification of 1,000X. Nuclear abnormalities were also recorded as cytotoxic parameters using the same magnification.

### Evaluation of non-specific innate immunity

#### Serum total protein and globulin

Serum total protein and albumin levels were determined spectrophotometrically using reagent kits purchased from Human Gesell Schaft fur Biochemical und Diagnostic GmbH, Germany. The absorbance was measured at a wavelength of 546 nm (within the range of 530–570 nm) (Henry 1974; Weichselbaum 1946). Blood serum globulin was calculated by subtracting the concentration of albumin from the total protein concentration (Coles 1986).

#### Phagocytic activity

Phagocytic activity was assessed using the EZCell™ Phagocytosis Assay Kit (Green zymosan, China). A series of green zymosan slurries, ranging from 0 to 4 µl, were added to a 96-well plate. The total volume was adjusted to 100 µl with phagocytosis buffer. After thorough mixing, the plate was analyzed using flow cytometry (FACSCalibur™, USA) and measured fluorometrically. The analysis was performed in the FL1 channel of the flow cytometer, which was equipped with a laser capable of excitation at 488 nm.

#### Serum lysozyme activity

Serum lysozyme activity was assessed using Rat ELISA kits from CUSABIO Co., China. Briefly, the serum samples from infected and non-infected fish were diluted 1:2000 before the test. In a 96-well assay plate, 50 µl of standard solution and sample were added, along with 50 µl of HRP-conjugate (1X) per well. The plate was then incubated for 60 min at 37ºC. Afterward, the plate was washed five times with wash buffer (200 µl) and allowed to stand for 2 min. Subsequently, 90 µl of 3,3',5,5'-Tetramethylbenzidine was added to each well and incubated for 20 min at 37ºC. Stop solution (50 µl) was added, gently mixed, and the optical density was measured at 450 nm using a Microplate Reader (Model MR 5000, Canada).

#### Nitric oxide production (NO)

The serum concentration of NO was determined spectrophotometrically in a microplate using the Griess reaction (Tarafder and Rathore 1988) and a NaNO2 standard curve at a wavelength of 540 nm.

### Statistical analysis

The data were statistically expressed as mean ± standard deviation (SD) using the SPSS program version 20. An unpaired t-test was performed, followed by a post hoc Duncan multiple range test to compare the groups at the 0.05 probability level.

## Results

### Prevalence of infection

Microscopic examinations of blood films revealed that 10 out of the total specimens (10%) were infected with *Trypanosoma* sp. Meanwhile, PCR estimated 15 samples (15%) as positive for *Trypanosoma*.

### Clinical examination

Ten fish exhibited one or more of the following signs depending on the stage of infection. The main signs included paleness in the mucous membranes and gills, which were the most frequently detected signs, along with emaciation and abdominal distension. Additionally, abnormal behavioral signs, such as imbalance and loss of the escape reflex, were observed. The liver appeared pale, and there was serosanguinous fluid in the abdominal cavity.

### Parasitological examination

**Fig. 1:**
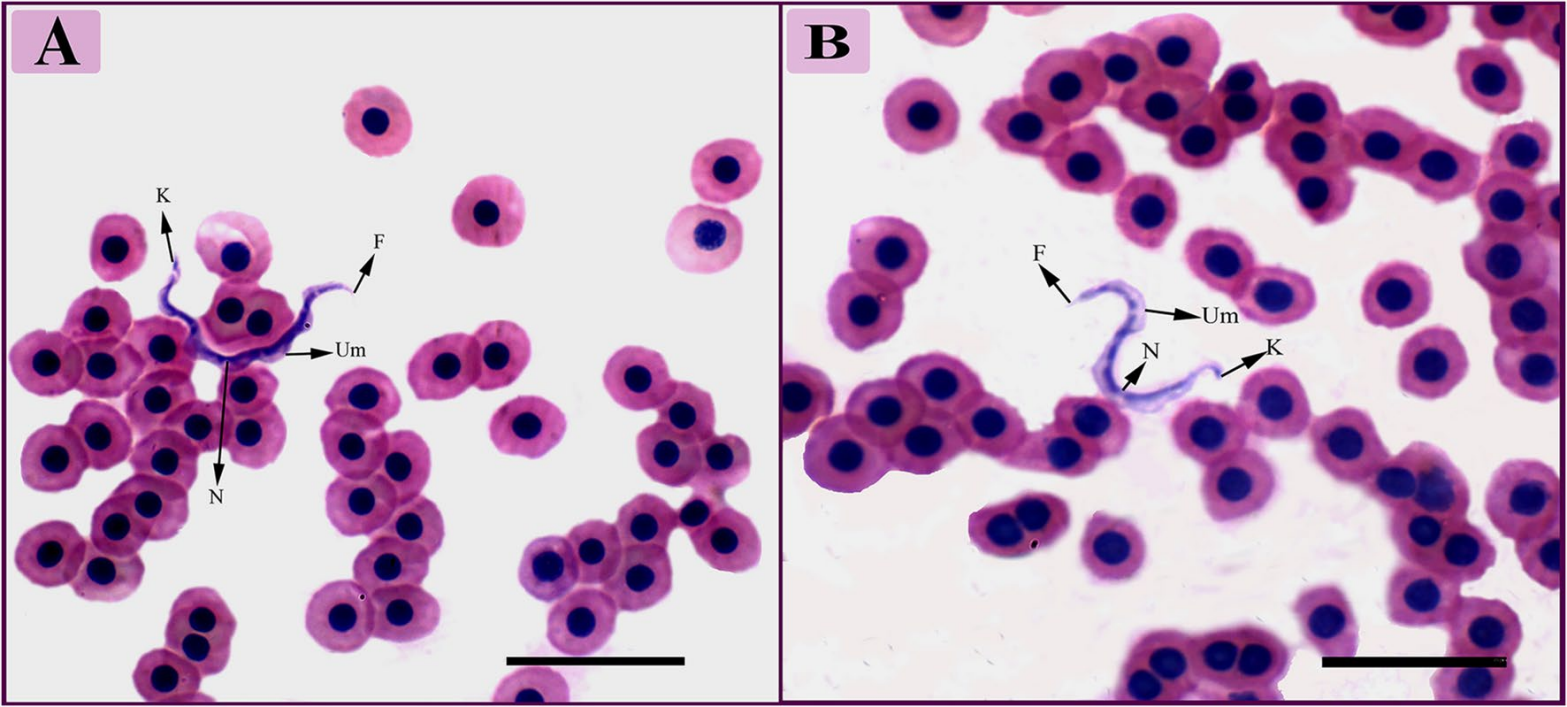
Trypomastigote forms in the blood film of African catfish, *Clarias gariepinus*, stained with hematoxylin and eosin (100X). N, nucleus; K, kinetoplast; Um, undulating membrane; F, flagellum

Under the light microscope, *Trypanosoma* appeared as active and wriggling trypanosomes, identified as *Trypanosoma mukasai* as described by Hussein et al. (2010) (Fig. 1). The morphological data revealed that the trypanosomes were elongated and cylindrical in shape, measuring between 67.80–82.79 µm in total length including the free flagellum (Table 1).

**Table 1:** Comparison of morphometric characteristics (in µm) between the present and previous studies on *Trypanosoma* sp. infecting *Clarias gariepinus*. Data are presented as mean ± SD

Measurements	The present study	El-Tantawy and El-Sherbiny (2010)	Smit et al. (2020)
Total length (TL) including free flagellum	73.36 ± 8.21 (67.80–82.79)	48.6 (41.8–62.7)	39.84 ± 6.96 (30.25–46.91)
Flagellum length (FL)	5.83 ± 1.49 (4.60–7.48)	10.0 (8.8–11.0)	–
Body width anterior (BWA)	1.97 ± 0.35 (1.60–2.30)	–	–
Body width nucleus (BWN)	2.86 ± 0.47 (2.57–3.40)	2.2	–
Body width posterior (BWP)	2.13 ± 0.15 (2.0–2.30)	–	–
Posterior pale region (PPR)	1.73 ± 0.15 (1.60–1.90)	–	–
Nuclear length (NL)	5.27 ± 0.21 (5.10–5.50)	4.6 (3.3–5.5)	–
Nuclear width (NW)	1.97 ± 0.58 (1.30–2.30)	2.1	–
Number of folds (NF)	6.00 ± 1.0 (5.0–7.0)	–	–
Distance from the posterior margin of the nucleus to the kinetoplast	33.63 ± 5.51 (27.60–38.40)	15.1 (13.2–18.7)	–
Distance from the anterior margin of the nucleus to the anterior end of the body	32.59 ± 9.64 (23.90–42.96)	18.9 (16.5–23.1)	–
Distance from the posterior margin of the nucleus to the posterior end of the body	36.06 ± 5.93 (29.80–41.60)	–	–
Nuclear index (NI)	1.107 ± 0.615 (0.96–1.25)	–	1.2

The parasites exhibited similar morphology overall. They had a tapered anterior end and a tapered or curled posterior end, with some cases showing a slightly rounded posterior. The cytoplasm appeared granulated, and normal oval or rectangular nuclei were observed (Fig. 1).

The nuclear indices (NI) ranged from 0.97–1.2, indicating that the nucleus was generally positioned centrally in the mid-posterior half of the body. The nucleus measured between 5.10–5.50 µm in length and 1.30–2.30 µm in width. The kinetoplast was typically deeply stained, small, rounded, and positioned a certain distance away from the posterior end. The undulating membrane was well-developed, with seven or fewer waves. The free flagellum was visible, short, and measured 4.60–7.48 µm in length (Table 1).

### Confirmation of *Trypanosoma* sp. Identity using PCR

The *Trypanosoma*-specific SSU rRNA gene was amplified, resulting in a 570 bp amplicon obtained from the positive samples (15 fish, 15%). No amplification products were detected in the no-template control and negative extraction control (data not shown).

## Cytotoxicity effect of trypanosome infection in erythrocytes and nuclear abnormalities

**Fig. 2:**
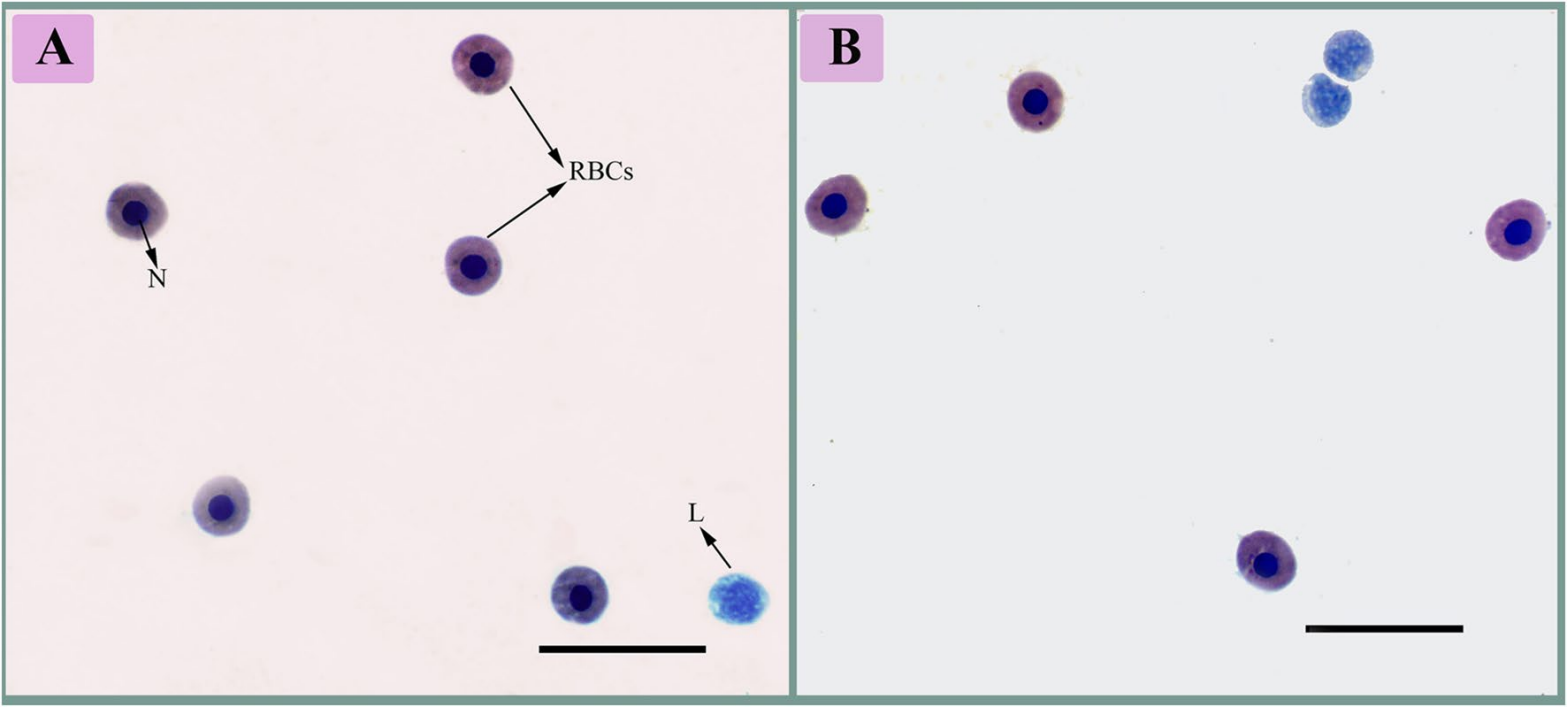
Blood film of the control African catfish, *Clarias gariepinus*, showing the normal rounded shape of the nucleated erythrocytes (RBCs), with a round nucleus (N) and lymphocytes (L), stained with Giemsa stain (100X)

**Fig. 3:**
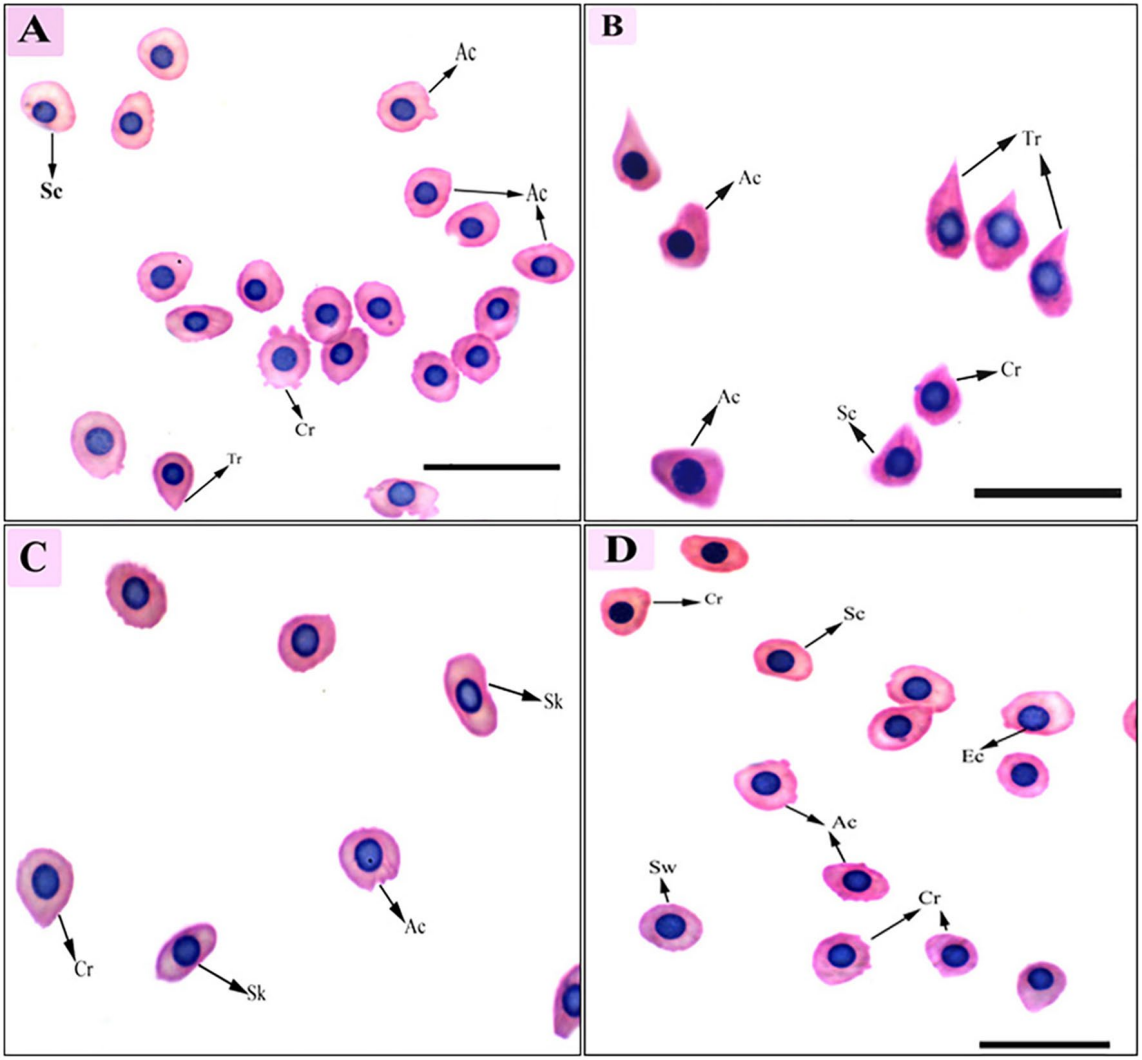
Blood film of African catfish infected with *Trypanosoma mukasai*, showing various alterations in erythrocytes. Ac, Acanthocytes; Cr, Crenated cells; Sk, Sickle cells; Sc, Schistocytes; Sw, Swollen cells; Tr, Tear-drop like cells; Ec, eccentric nucleus. Stained with hematoxylin and eosin (100X)

As shown in Fig. 2, the blood film from the non-infected fish displayed the normal structure of catfish erythrocytes, which are oval-shaped with a central nucleus. In contrast, the infected fish exhibited various morphological changes in their erythrocytes (Fig. 3). One of the major alterations observed was the appearance of acanthocytes, where the erythrocytes had fewer projections on their surface (Fig. 3a-d).

**Fig. 4:**
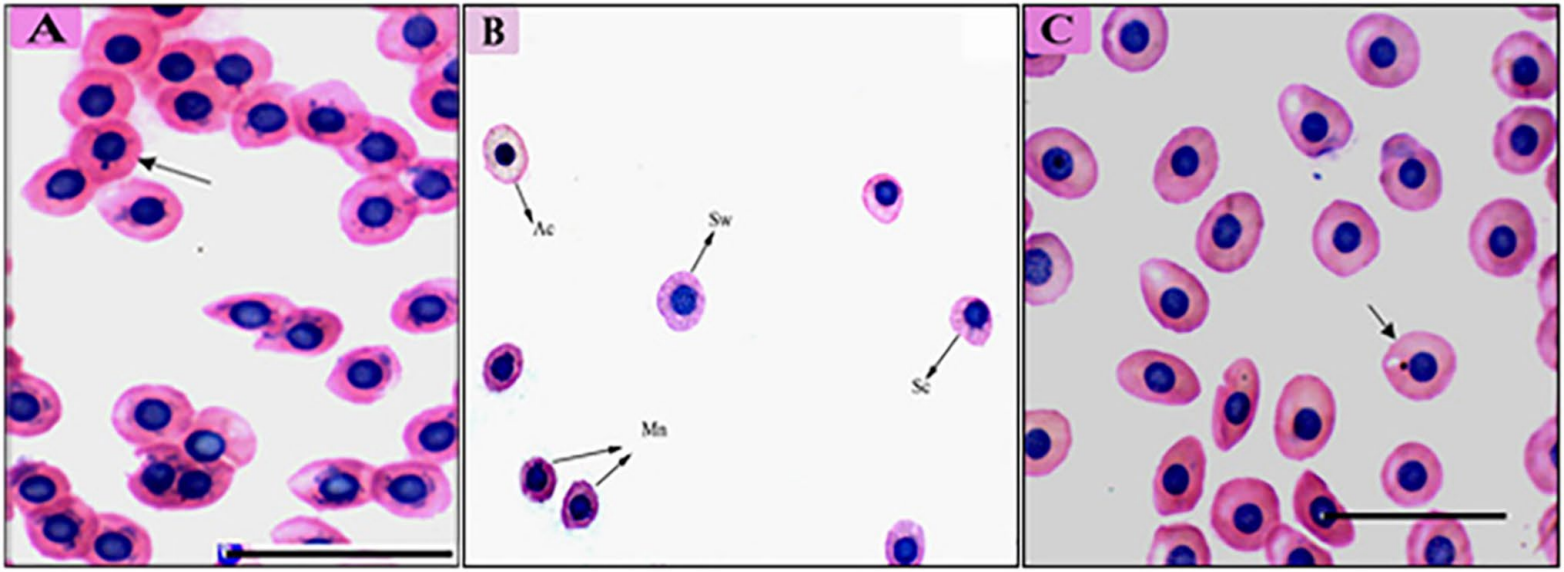
Blood film of African catfish, *Clarias gariepinus*, infected with *Trypanosoma mukasai*, showing the presence of micronuclei frequencies (arrow). Ac, Acanthocytes; Sc, Schistocytes; Sw, Swollen cells; Mn, micronuclei. Stained with hematoxylin and eosin (100X)

Several other morphological changes were recorded in the erythrocytes. Echinocytes or crenated cells were observed, characterized by an irregular cell surface with numerous projections (Fig. 3). Schistocytes or schizocytes were also present, displaying erythrocytes with fragmented parts and two pointed ends (Fig. 3a, b, and d). Teardrop-like cells, resembling a tear with pointed apices, were observed (Fig. 3b). Additionally, sickle cells and swollen cells were identified (Fig. 3c and d). Nuclear abnormalities were also recorded, including fragmented nuclei and eccentric nuclei (Fig. 3d). DNA damage led to the fragmentation of cell nuclei, forming micronucleus abnormalities (Fig. 4).

Table 2 presents a significant increase of 54.57% in the frequency of erythrocyte abnormalities and a 1.3% significant increase in micronuclei in infected fish compared to non-infected ones.

**Table 2:** Percentage of altered erythrocytes mean ± SD (range) % per 100 cells in control and infected *Clarias gariepinus*

Groups	Control (*n *= 5)	Infected (*n* = 4)
Altered RBCs (%)	2.63 ± 0.513 (2.2–3.2)	54.57 ± 3.37 (50.68–56.66)
Micronuclei (%)	0.37 ± 0.64 (0–1.1)	1.3 ± 0.65 (0.6–1.9)

### Immunological parameters

**Fig. 5:**
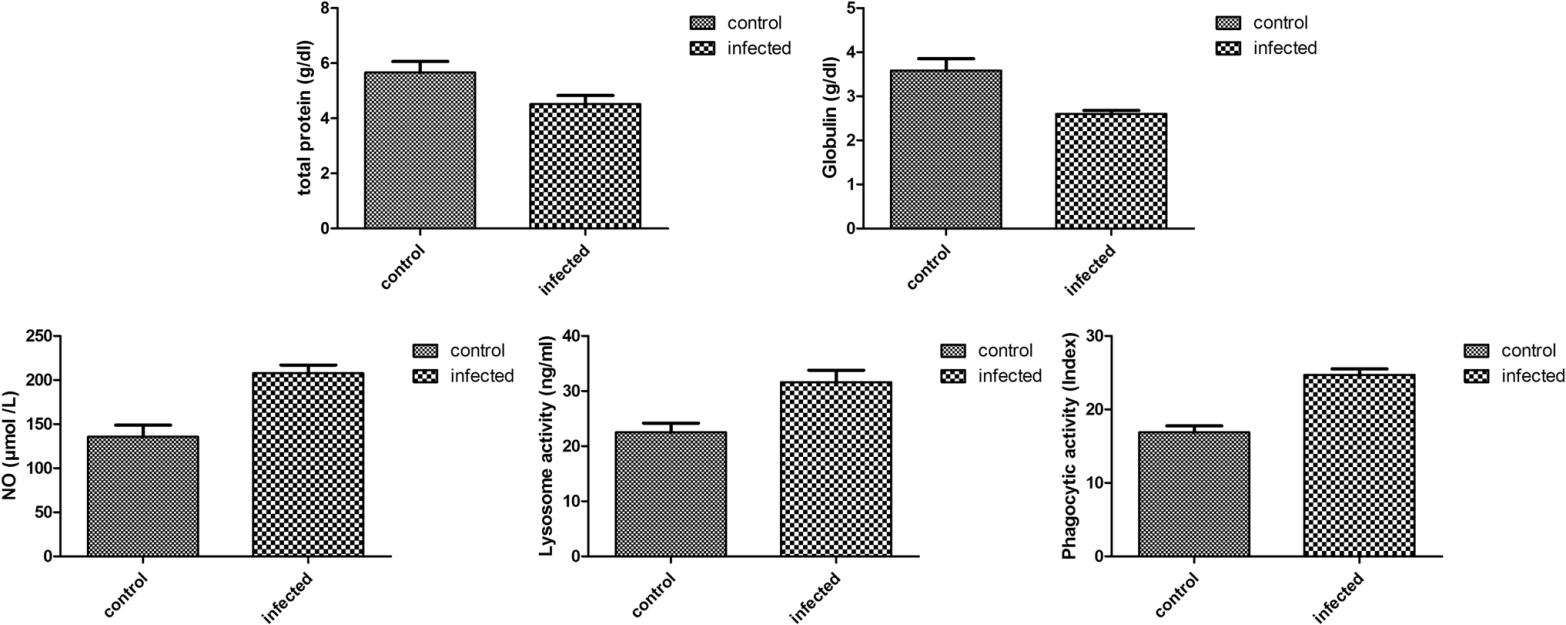
Levels of different innate immune parameters (serum total protein, serum globulin, nitric oxide (NO), serum lysozyme activity, and phagocytic index) in fish infected with *Trypanosoma* sp. compared to non-infected fish

The infected fish exhibited a non-significant decrease in serum total protein (*P* = 0.09, Fig. 5), while there was a statistically significant reduction in globulin levels (*P* = 0.026, Fig. 5) compared to the non-infected fish. Additionally, humoral immune parameters such as NO production and lysozyme activity showed a statistically significant increase (*P* = 0.011 and 0.03 for NO and lysozyme activity, respectively, Fig. 5) in the infected fish compared to the non-infected fish. Similarly, a highly significant increase in phagocytic activity (*P* = 0.0028, Fig. 5) was recorded in the serum of the infected fish compared to the non-infected fish.

## Discussion

Trypanosomes*,* which are hemoflagellate protozoan parasites, are prevalent in freshwater fishes and various terrestrial vertebrates, while nvertebrates such as leeches and tsetse flies play a crucial role in their transmission (Biswas et al. 2001; Ortiz-Baez et al. 2020). Natural infections with *Trypanosoma* can cause abnormalities in hematological parameters (Fujimoto et al. 2013). This study specifically focuses on the effects of trypanosome infection as a blood parasite on erythrocyte alterations and non-specific serum immunological parameters. The prevalence of *Trypanosoma* sp. infection was found to be 15%, which is lower than the rate reported by Okoye Uzodinma et al. (2016) in Nigeria for the same fish species. In South Africa, the incidence of *C. gariepinus* infection with *T. mukasai* was 19%, using blood smear technique (Ferreira and Avenant-Oldewage 2013).

Freshwater fish *Trypanosoma* have been identified by Woo and Black (1984) and Lom and Dyková (1992) using either morphological criteria or host range. The morphology of *Trypanosoma* can vary widely across different hosts, making measurements the most crucial quantitative morphological characteristics. Other techniques, such as molecular cloning and sequencing, are more concise, especially in cases of mixed infection; however, they demand increased laboratory work and incur higher costs.

Our results revealed *Trypanosoma* infection in the blood of *C. gariepinus*, morphologically identified as *Trypanosoma musakai*, which aligns with previous studies (El-Tantawy and El-Sherbiny 2010; Hussein et al. 2010). Abdel Mawla et al (2018) also reported the same species in the same host and geographic area. However, to the best of our knowledge, molecular identification of *Trypanosoma* infection in freshwater fish is scarce. This scarcity could be attributed to the intensity of the infection and the challenges of multiplying the parasite in experimental animals. Smit et al. (2004) observed variations in the morphometric analysis of all detected *T. mukasai*. Consequently, PCR has been utilized for direct parasite detection and identification, offering enhanced sensitivity and reliability. PCR techniques have significantly improved the efficiency of identifying infections, particularly in cases of mixed trypanosome infections (Woolhouse et al. 1996). This technique can be carried out using real-time PCR with *Trypanosoma* species-specific DNA probes (Gibson et al. 1988) or conventional PCR with *Trypanosoma* species-specific DNA primers (Habeeb et al. 2021). That being said, nested PCR or other more confirmatory methods should be considered to improve diagnostic accuracy.

Hemoflagellate parasites have captured the attention of molecular biologists, immunologists, and geneticists as intriguing models for studying various biological phenomena. However, the induction of DNA damage with parasitic diseases in catfish, as a model for toxicology, has yet to be investigated.

Our results revealed various malformations in the erythrocytes and identified nuclear abnormalities associated with trypanosome infection. Acanthocytes, crenated cells, schistocytes, swollen cells, and teardrop alterations were frequently observed in the blood of infected fish. Additionally, to the best of our knowledge, this study represents the first recorded occurrence of micronuclei associated with *Trypanosoma* infection.

Variations in the types of malformations depend on the physiological adaptation, fish activity, cell size, and other environmental factors that can affect the osmotic fragility of the cell (Gupta and Gupta 2012). In a study by Islam and Woo (1991), it was reported that the excretion of hemolysin, which is associated with acidosis and hemodilution, was responsible for anemia correlated with trypanosome infection in goldfish.

Shahi et al. (2013) reported the presence of various abnormal red blood cells in trypanosome-infected fish. Additionally, in trypanosome-infected fish, nuclear fragments, basket cells, and cell casts were observed (Joshi and Dabral 1981). Gupta and Gupta (2012) documented vacuolation, karyorrhexis, cytolysis, and cell death in the erythrocytes of infected *Clarias batrachus*. Moreover, infected fish may experience a decreased lifespan of red blood cells due to erythropenia (Gupta and Gupta 2012).

Evidence from research suggests that certain trypanosomes produce soluble materials capable of causing minimal lysis due to the rapid mobility of flagellates in the circulatory system, leading to hemolysis (Gupta and Gupta 2012). Reductions in total erythrocyte counts were reported in *C. batrachus* infected with trypanosomes (Joshi and Dabral 1981). This decrease in total erythrocyte count values indicates that the parasites are affecting erythropoiesis (Burgert et al. 2020), leading to a decline in the blood's oxygen-carrying capacity, inhibition of normal erythrocyte production, and increased production of distorted cells (Gupta and Gupta 2012). Consequently, the distortion of erythrocytes serves as a reliable indicator of DNA damage. Moreover, parasite-released toxic factors can increase erythrocyte phagocytosis in the spleen (Maegraith 1969).

The immune system plays a crucial role in protecting against infections and maintaining internal homeostasis. It consists of two components: innate immunity and adaptive immunity. Innate immunity acts as the host's initial defense line against microbial invasions, while adaptive immunity plays a vital role in protecting against recurrent infections (Secombes and Belmonte 2016). Fish, being one of the earliest evolved organisms, primarily rely on innate immunity to combat a wide range of pathogens (Sahoo 2006). In this study, various essential aspects of innate immunity, such as phagocytic activity, NO production, and lysozyme, were investigated.

In our study, we observed a significant increase in phagocytic activity in infected fish compared to non-infected fish. This increase may be attributed to the release of trypanosome antigens, which can enhance various immunochemical strategies, including boosting phagocyte proliferation, stimulation, and complement activation (Igbokwe 1994). One of the key trypanosome antigens responsible for activating macrophages is glycosylphosphatidylinositol, which serves as the membrane anchor for variant surface glycoproteins (Tachado et al. 1999). Consequently, classically-activated macrophages have been shown to play a role in clearing the parasite through the process of phagocytosis (Shi et al. 2004). Additionally, the trypanosome bloodstream sialidase enzyme can induce surface changes in red blood cells, leading to subsequent phagocytosis (Buratai et al. 2006).

Cytokine-activated macrophages produce a high amount of nitric oxide (NO), which is consistent with the results of our study. The elevated level of NO in infected fish may be attributed to the increased expression of type II NO synthase (NOS-II), which generates NO and kills the parasite (Gobert et al. 2000). Furthermore, as a result of activation of mononuclear phagocytes, particularly macrophages, lysozyme is synthesized and released into the blood (Kokoshis and Di Luzio 1979). Following antigenic stimulation of the immune system, there is a significant increase in serum lysozyme levels (Maraghi et al. 2012).

Moreover, trypanosomiasis can cause hepatocellular damage (e.g., hepatic necrosis, vacuolar degeneration, and dilated blood vessels) (Aly et al. 2005), slightly increase capillary permeability for plasma proteins, and secrete proteases into the bloodstream of infected hosts (Troeberg et al. 1996), leading to protein degradation (Stoskoph 1993). Therefore, as a result of the immune system activation mentioned above, *Trypanosoma* sp. can decrease the levels of serum total protein and globulins.

Parasitic diseases pose a significant problem for fisheries, hampering productivity in both wild and cultured fish populations (Subasinghe and Philips 2002). These diseases infect various groups of fish and cause considerable damage to their hosts, weakening the immune system and increasing susceptibility to secondary infections. Moreover, they disrupt the normal physiological conditions of fish, leading to nutritional deficiencies and, in some cases, mass mortalities, resulting in economic losses (Balarin 1985).

## Conclusions

We conclude that trypanosome infection has a significant impact on blood parameters, particularly red blood cells. This study observed various morphological and nuclear abnormalities in the red blood cells of *C. gariepinus*, indicating these alterations as potential cytotoxicity biomarkers in wild fish. Additionally, *Trypanosoma* sp. demonstrated a pronounced effect on non-specific innate and humoral immunity, leading to elevated levels of NO, lysozyme activity, and phagocytic activity.

## Data Availability

Data & materials are available upon reasonable request.
